# High-Density Lipoprotein from Chronic Kidney Disease Patients Modulates Polymorphonuclear Leukocytes

**DOI:** 10.3390/toxins11020073

**Published:** 2019-02-01

**Authors:** Jana Raupachova, Chantal Kopecky, Gerald Cohen

**Affiliations:** 1 Department of Nephrology and Dialysis, Medical University of Vienna, Vienna A-1090, Austria; jraupach@gmx.at; 2 School of Medical Sciences, Faculty of Medicine, University of New South Wales, Sydney, NSW 2052, Australia; c.kopecky@unsw.edu.au

**Keywords:** high-density lipoprotein, polymorphonuclear leukocytes, apoptosis, CD11b, immunology, inflammation, signal transduction

## Abstract

The anti-inflammatory properties of high-density lipoproteins (HDL) are lost in uremia. These HDL may show pro-inflammatory features partially as a result of changed protein composition. Alterations of polymorphonuclear leukocytes (PMNLs) in chronic kidney disease (CKD) may contribute to chronic inflammation and high vascular risk. We investigated if HDL from uremic patients is related to systemic inflammation by interfering with PMNL function. PMNL apoptosis was investigated by assessing morphological features and DNA content. CD11b surface expression was quantified by flow cytometry. Oxidative burst was measured via cytochrome c reduction assay. Chemotaxis was assessed by using an under-agarose migration assay. We found that HDL from CKD and hemodialysis (HD) patients significantly attenuated PMNL apoptosis, whereas HDL isolated from healthy subjects had no effect on PMNL apoptosis. The use of signal transduction inhibitors indicated that uremic HDL exerts anti-apoptotic effects by activating pathways involving phosphoinositide 3-kinase and extracellular-signal regulated kinase. Healthy HDL attenuated the surface expression of CD11b, whereas HDL from CKD and HD patients had no effect. All tested isolates increased the stimulation of oxidative burst, but did not affect PMNL chemotactic movement. In conclusion, HDL may contribute to the systemic inflammation in uremic patients by modulating PMNL functions.

## 1. Introduction

High levels of high-density lipoproteins (HDL) are associated with decreased cardiovascular risk [1] related to its diverse biological functions including the efflux of cholesterol from macrophages [2] and potent anti-inflammatory properties [3,4]. However, the concept of therapeutically increasing HDL levels failed to protect against cardiovascular events [5]. In recent years it has become evident that in inflammatory diseases such as coronary artery disease, chronic kidney disease (CKD), diabetes, and rheumatoid arthritis HDL is qualitatively altered and loses its anti-inflammatory properties [6,7,8].

Studies on the effect of HDL isolated from hemodialysis (HD) patients on vascular smooth muscle cells [9] and monocytes and dendritic cells [10] found reduced or abolished anti-inflammatory properties. However, the effect of HDL from CKD and HD patients on polymorphonuclear leukocytes (PMNLs), which have binding sites for HDL [11] and its major apolipoprotein constituent ApoA-I [12] has not yet to be investigated.

PMNLs play a key role in the nonspecific immune defense against bacterial infections. After the chemotactic movement to the site of infection, they first ingest the microorganism by phagocytosis and then use reactive oxygen metabolites and proteolytic enzymes to kill it. Any disturbance of these essential PMNL functions leads to an increased risk of infection [13]. In turn, pre-activation and priming of PMNLs in the course of inflammatory diseases is characterized by increased expression and activation of inflammatory markers such as CD11b, which represents an atherosclerotic risk marker in patients with a disturbed lipid metabolism [14]. Thus, the organized elimination of inflammatory PMNLs through apoptotic cell death is critical to avoid unnecessary inflammation [15].

The aim of this study was to investigate if modulation of PMNLs by HDL contributes to systemic inflammatory characteristics of uremic patients. To this end, the effect of HDL from CKD and HD patients on various PMNL functions was determined by in vitro assays and compared to the effect of HDL from healthy subjects (HS-HDL). We found that HDL from uremic patients significantly differs from HS-HDL; it decreased spontaneous PMNL apoptosis but had no attenuating effect on the surface expression of CD11b.

## 2. Results

### 2.1. Clinical Characteristics of Study Participants

The clinical parameters and characteristics of the study participants are shown in Table 1 and Table 2. HDL isolated from stage 3 CKD patients and HDL isolated from stage 4 CKD patients did not show any statistical difference in their biological effects tested in this study (data not shown). Therefore, the combined data of these groups of patients are shown. Hemodialysis (HD) patients were dialyzed on standard bicarbonate basis for 4 to 5 h three times a week with biocompatible polysulfone HD membranes (Fresenius, Oberursel, Germany). The Kt/V values were 1.2 in all patients. None of the HD patients had residual renal function. The underlying diseases in the CKD group were vascular nephropathy (4), (CPI, immune complex, pauci immun) glomerulonephritis (3), diabetic nephropathy (2), cystic kidney (2), pyelonephritis (1), and lupus nephritis (1). The underlying diseases in the HD group were pyelonephritis (3), diabetic nephropathy (2), (chronic, IgA) glomerulonephritis (2), vascular nephropathy (1), renal agenesis (1), and hypertensive nephropathy (1). The rest were of unknown origin.

### 2.2. Polymorphonuclear Leukocyte Apoptosis

**Figure 1:**
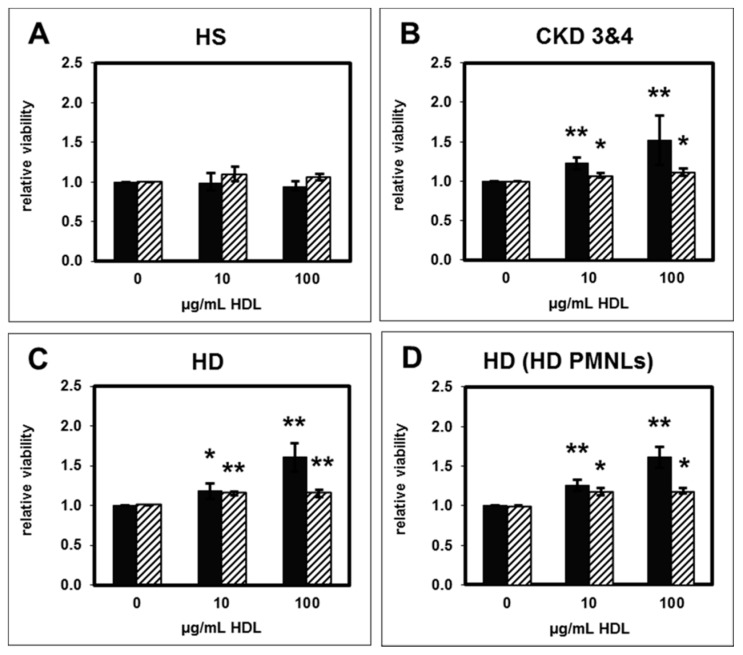
Effect of (**A**) HS-HDL, (**B**) CKD3 and 4-HDL, (**C**) HD-HDL on apoptosis of PMNLs isolated from healthy subjects and the effect of (**D**) HD-HDL on apoptosis of PMNLs from HD patients (HD; HD PMNLs). Apoptosis was determined by assessing morphological features (black bars) and by measuring DNA content (striped bars). Data presented as relative viability normalized to the value for PMNLs without HDL. (**A**,**B**) *n* = 15 for 100 µg/mL; *n* = 9 for 10 µg/mL. (**C**) *n* = 16. (**D**) *n* = 9. * *p* < 0.05 and ** *p* < 0.01 vs. 0 µg/mL HDL. Data shown are mean values ± SEM. HS, healthy subjects; CKD, chronic kidney disease; HD, hemodialysis; PMNLs, polymorphonuclear leukocytes.

HS-HDL had no significant effect on PMNL apoptosis as assessed by evaluating morphological criteria and by measuring DNA content (Figure 1A). PMNL viability in the absence of HDL was normalized to 1 (“viability factor”). The absolute percentage of viable PMNLs was 23 ± 2% (morphology) and 32 ± 3% (DNA content). This finding is consistent with the literature reporting that DNA fragmentation is a much later event in apoptosis compared to morphological changes [16]. Therefore, measuring the DNA content resulted in a higher percentage of viable cells.

HDL from patients with CKD stage 3 and 4 significantly reduced PMNL apoptosis and thereby increased the percentage of viable PMNLs (Figure 1B), a characteristic pro-inflammatory behavior. PMNL apoptosis was also attenuated by HDL from HD patients (Figure 1C).

Apoptosis of PMNLs isolated from HD patients was significantly reduced by incubation with HD-HDL (Figure 1D). There was no significant difference between the effect of HD-HDL on PMNLs from healthy subjects and from HD patients, demonstrating that exposure of the PMNLs to the uremic milieu did not attenuate the anti-apoptotic function of HD-HDL.

**Figure 2:**
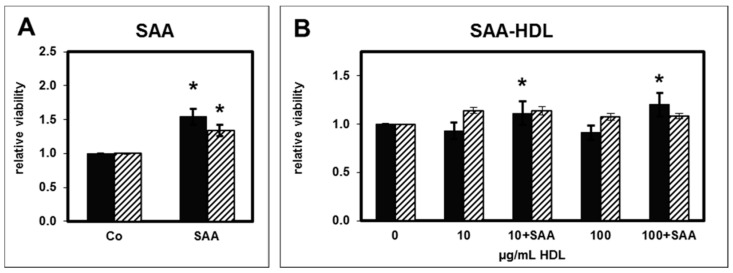
Effect of serum amyloid A (SAA) at a final concentration of 10 µg/mL (**A**; *n* = 4) and of HS-HDL spiked with SAA at final concentrations of 10 µg/mL and of 100 µg/mL (**B**; *n* = 8) on apoptosis of PMNLs from healthy subjects. Black bars: Apoptosis determined by assessing morphological features; striped bars: by measuring the DNA content. Data presented as relative viability normalized to the value for PMNLs without SAA (Co: buffer as control, 0 µg/mL HDL). *n* = 4. * *p* < 0.05 vs. Co, 0 µg/mL HDL; data are shown in mean values ± SEM.

The acute phase protein serum amyloid A (SAA) has previously been shown to be enriched in HD-HDL [10]. SAA induced the expression of inflammatory cytokines in human monocytes [10]. When exposed to SAA, we observed a significant reduction in PMNL apoptosis to a similar extent as for CKD-HDL and HD-HDL (Figure 2A). This is in agreement with results obtained by El Kebir et al. [17].

It was previously shown that incorporation of SAA in HDL from healthy individuals reverses the anti-inflammatory effect of HDL [10]. Therefore, we tested the effect of HS-HDL that was spiked with SAA (SAA-HDL) (Figure 2B). Whereas SAA-HDL showed a slight decrease in PMNL apoptosis determined by assessing morphological features, there was no difference when using DNA content to measure apoptosis.

**Figure 3:**
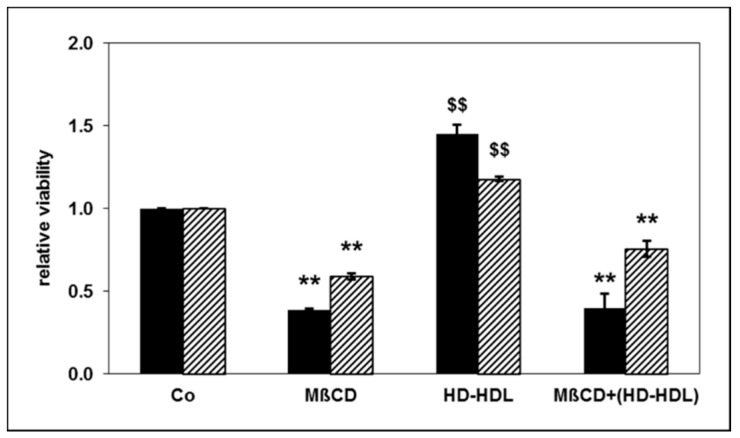
Effect of methyl-β-cyclodextrin (MβCD) at a final concentration of 3 mg/mL and of HD-HDL at a final concentration of 100 µg/mL on apoptosis of PMNLs from healthy subjects. Apoptosis was determined by assessing morphological features (black bars) and by measuring the DNA content (striped bars). The data are presented as relative viability: The value for PMNLs without MβCD and HD-HDL (Co: buffer as control) was set as viability factor 1.00: *n* = 4. ** *p* < 0.01 vs. absence of MβCD, ^$$^ *p* < 0.01 HD-HDL vs. the absence of HDL; data are shown in mean values ± SEM.

HDL has been suggested to alter cellular functions by lowering membrane cholesterol content, especially within lipid rafts [18]. We investigated the effect of selective lipid raft destruction on PMNL apoptosis using methyl-β-cyclodextrin (MβCD) to disintegrate lipid rafts [19]. MβCD treatment significantly increased PMNL apoptosis both alone and in the presence of apoptosis attenuating HD-HDL (Figure 3).

**Figure 4:**
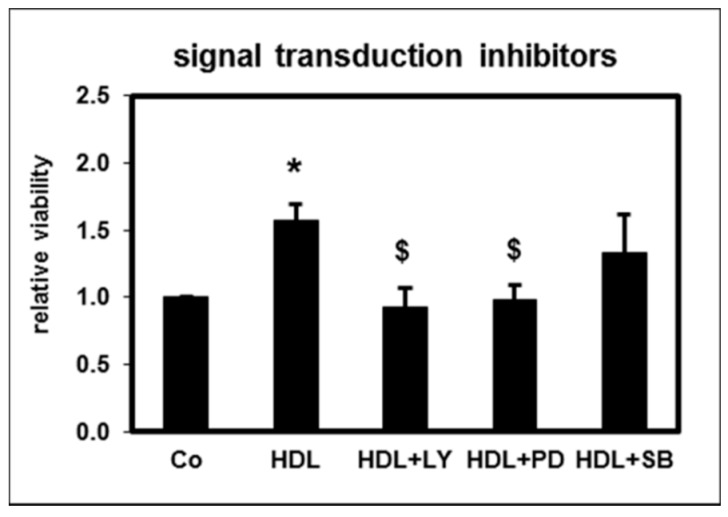
Effect of HD-HDL on apoptosis of PMNLs from healthy subjects in the presence of signal transduction inhibitors as assessed by morphological features. LY (LY294002; final concentration: 50 µM): inhibitor of phosphor-inositide 3-kinase; PD (PD98059; final concentration: 50 µM): inhibits p44/42 (extracellular-signal regulated kinase; ERK) by inhibiting MEK1,2; SB (SB203580; final concentration: 30 µM): inhibitor of p38 mitogen-activated protein kinase (MAPK). Data presented as relative viability normalized to the value for PMNLs with buffer control (Co: buffer as control). *n* = 8. * *p* < 0.05 vs. Co; ^$^ *p* < 0.05 vs. HDL; data are shown in mean values ± SEM.

To elucidate the signaling pathways related to the anti- apoptotic effect of HD-HDL, we used specific inhibitors of phosphoinositol 3-kinase (PI3K), p44/42 (ERK) and p38 MAPK. Whereas the inhibition of PI3K and ERK completely abolished the HDL effect on apoptosis, inhibition of p38 MAPK had no significant impact (Figure 4). These data indicate that HD-HDL exerts anti-apoptotic effects by activating signal transduction pathways involving PI3K and ERK.

### 2.3. CD11b Surface Expression

**Figure 5:**
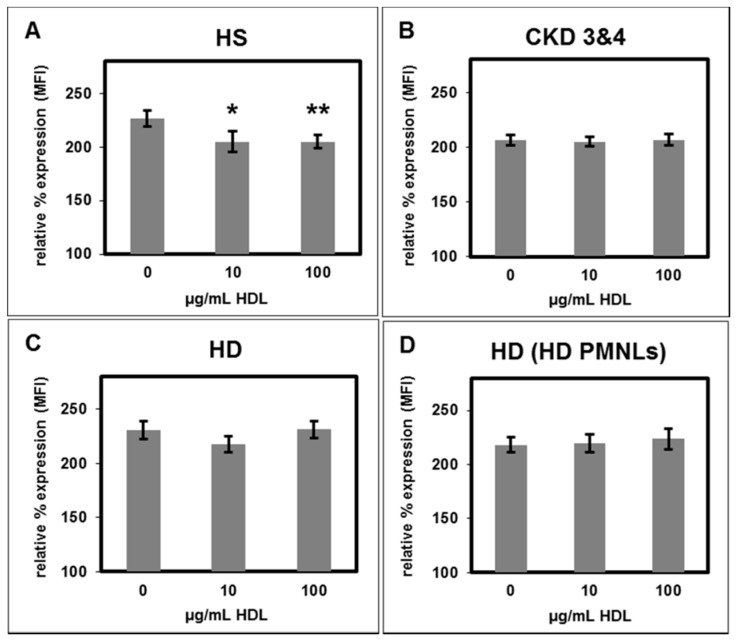
CD11b surface expression stimulated by N-formyl-methionyl-leucyl-phenylalanine (fMLP). Effect of HDL from (**A**) healthy subjects, *n* = 7; (**B**) CKD patients stage 3 and 4, *n* = 16; (**C**) HD patients, *n* = 7; on PMNLs from healthy subjects; (**D**) effect of HDL from HD patients on PMNLs from HD patients, *n* = 9; the unstimulated mean fluorescence intensity (MFI) value in the absence of HDL was set as 100%, * *p* < 0.05 and ** *p* < 0.01 vs. 0 µg/mL HDL; data shown are mean values ± SEM.

In patients with a disturbed lipid metabolism, increased PMNL surface expression of CD11b is related to an increased atherosclerotic risk. We measured CD11b surface expression by flow cytometry using a fluorescence labelled anti-CD11b antibody. HS-HDL attenuated N-formyl-methionyl-leucyl-phenylalanine (fMLP)- stimulated CD11b surface expression on PMNL isolated from healthy subjects (Figure 5A). In contrast, treatment with CKD-HDL or HD-HDL had no effect on fMLP- stimulated CD11b surface expression (Figure 5B,C). Similarly, HD-HDL did not reduce the fMLP- stimulated CD11b surface expression on PMNLs isolated from from HD patients (Figure 5D).

**Figure 6:**
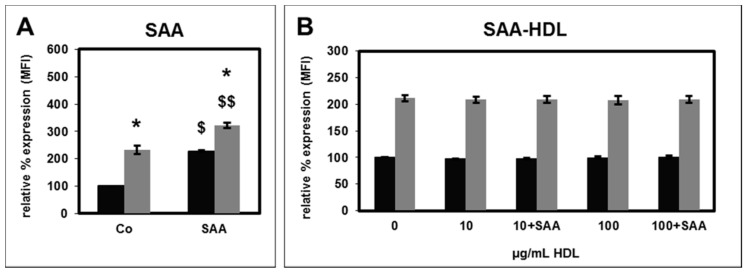
Effect of serum amyloid A protein (SAA) at a final concentration of 10 µg/mL (**A**; *n* = 3) and of HS-HDL and HS-HDL spiked with SAA at final concentrations of 10 µg/mL and of 100 µg/mL (**B**; *n* = 8) on the basal (black bars) and fMLP-stimulated (grey bars) CD11b surface expression. The mean fluorescence intensity (MFI) value for PMNLs without SAA (Co: buffer as control, 0 µg/mL HDL) was set as 100%. ^$^ *p* < 0.05 and ^$$^ *p* < 0.01 vs. control (Co: 0.01% BSA); * *p* < 0.05 vs. the unstimulated value; data shown are mean values ± SEM.

Whilst treatment of PMNL with SAA increased both basal and fMLP stimulated CD11b surface expression (Figure 6A), SAA-HDL had no effect (Figure 6B).

**Figure 7:**
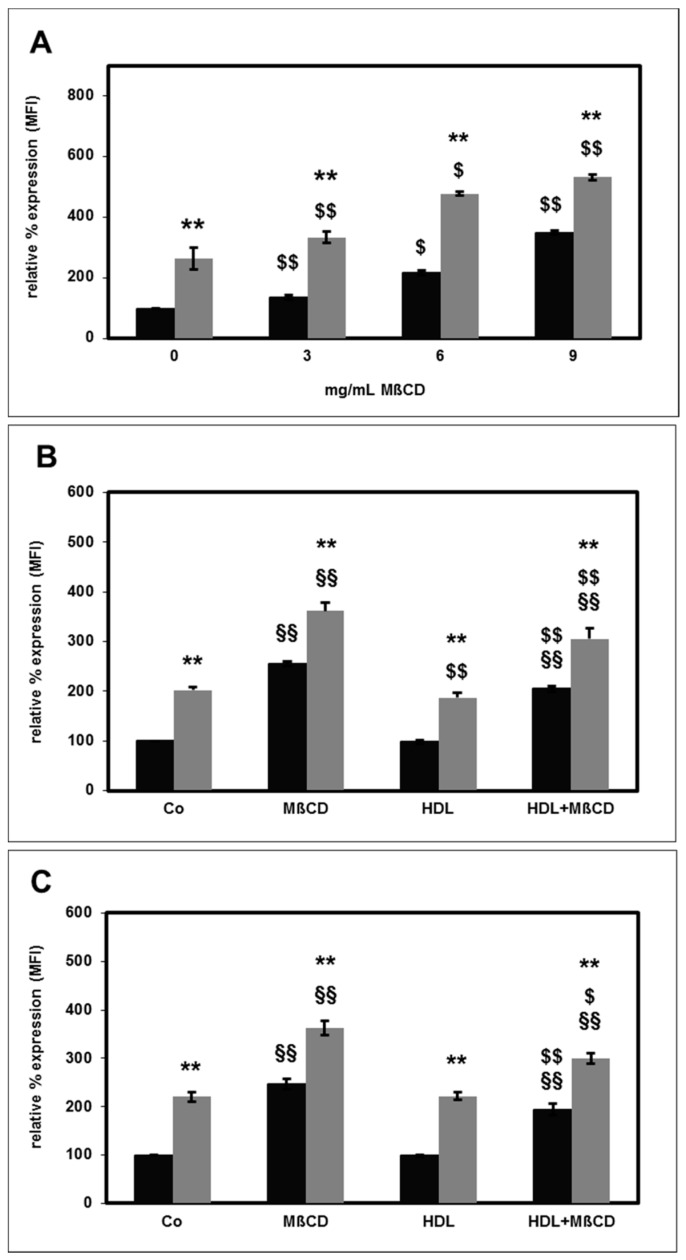
Effect of MβCD on basal (black bars) and fMLP-stimulated (grey bars) CD11b surface expression. The unstimulated CD11b expression measured as mean fluorescence intensity (MFI) in the absence of MβCD was set as 100%. Concentration dependency (**A**): *n* = 5 for 0 and 3 mg/mL MβCD; *n* = 3 for 6 and 9 mg/mL MβCD. Effect of MβCD (M; 6 mg/mL) in the absence and presence of 100 µg/mL HS-HDL (**B**; *n* = 4) and HD-HDL (**C**; *n* = 5). ** *p* < 0.01 vs. the unstimulated values; ^§^ *p* < 0.05 and ^§§^ *p* < 0.01 vs. the absence of MβCD; ^$^ *p* < 0.05 and ^$$^ *p* < 0.01 vs. the absence of HDL; data shown are mean values ± SEM.

Next, we assessed the influence of HDL on CD11b surface expression in the course of lipid raft disruption. MβCD significantly increased basal and fMLP-stimulated CD11b surface expression in a concentration dependent manner (Figure 7A). This effect was also observed in the presence of HS-HDL (Figure 7B) or HD-HDL (Figure 7C).

### 2.4. Oxidative Burst

**Figure 8:**
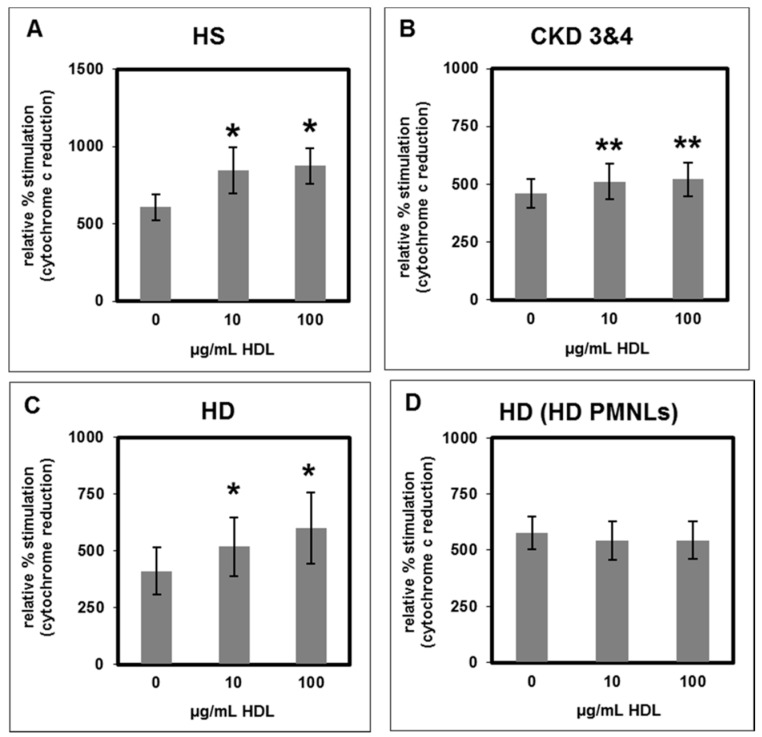
Influence of HDL on fMLP- stimulated oxidative burst. PMNLs isolated from healthy subjects treated with HDL from (**A**) healthy subjects, *n* = 6 for 10 µg/mL and *n* = 13 for 100 µg/mL; (**B**) CKD patients stage 3 and 4, *n* = 16; (**C**) HD patients, *n* = 8 for 10 µg/mL and *n* = 14 for 100 µg/mL; (**D**) effect of HDL from HD patients on PMNLs isolated from HD patients, *n* = 9; the unstimulated oxidative burst in the absence of HDL was set as 100%, * *p* < 0.05 and ** *p* < 0.01 vs. 0 µg/mL HDL; mean value ± SEM.

fMLP stimulated oxidative burst in PMNLs isolated from healthy subjects was increased by HS-HDL, CKD-HDL and HD-HDL to a similar magnitude (Figure 8A–C) whereas the basal levels were not affected (data not shown). Treatment of PMNLs isolated from HD patients with HD-HDL did not increase the stimulation of the oxidative burst (Figure 8D).

**Figure 9:**
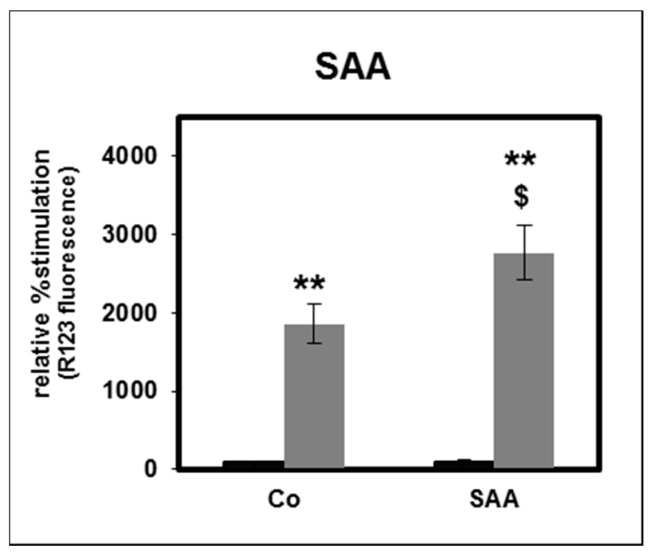
Effect of serum amyloid A protein (SAA) at a final concentration of 10 µg/mL (*n* = 5) on the basal (black bars) and fMLP-stimulated (grey bars) oxidative burst. The rhodamine 123 (R123) fluorescence of PMNLs without SAA (Co: buffer as control) was set as 100%. ^$^ *p* < 0.05 vs. control (Co); ** *p* < 0.01 vs. the unstimulated value; data are mean values ± SEM.

Whereas SAA treatment did not affect basal oxidative burst, SAA significantly increased fMLP-stimulated oxidative burst (Figure 9). As HS-HDL is not enriched with SAA, the stimulatory effect of HDL (Figure 8A–C) is not due to SAA. However, HS-HDL spiked with SAA did not increase fMLP-stimulated oxidative burst (data not shown).

**Figure 10:**
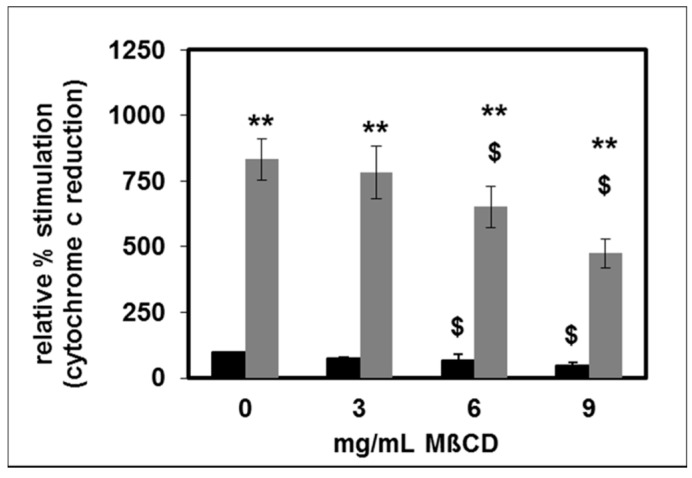
Effect of MβCD on basal (black bars) and fMLP- stimulated (grey bars) oxidative burst; *n* = 5 for 0 and 3 mg/mL MβCD; *n* = 3 for 6 and 9 mg/mL MβCD; the unstimulated oxidative burst in the absence of MβCD was set as 100%, ** *p* < 0.01 vs. unstimulated; ^$^ *p* < 0.05 vs. 0 mg/mL MβCD; mean values ± SEM.

Destruction of lipid rafts by MβCD significantly reduced both basal and fMLP-stimulated oxidative burst in a dose dependent manner (Figure 10).

## 3. Discussion

In this study, we have shown that HDL from CKD and HD patients significantly attenuated PMNL apoptosis, whereas HDL from healthy subjects did not affect PMNL apoptosis. We further found that HDL isolated from healthy subjects attenuated the stimulated surface expression of CD11b, whilst HDL from CKD and HD patients had no such impact.

In addition to the classical role in reverse cholesterol transport [2], it has become evident that HDL exerts several important biological functions [20]. HDL has potent anti-inflammatory, anti-oxidative and anti-thrombotic effects [21], which contribute to both cardio-protection [22], and immuno-regulation [23,24]. Animal experiments suggest that HDL can counteract inflammasome activation which enhances atherosclerosis [25]. Moreover, it has been shown that reconstituted HDL (rHDL) inhibits the activation of PMNLs and monocytes by phytohaemagglutinin (PHA) [26]. Isolated HDL also exerts anti-inflammatory functions on human monocytes by inhibiting CD11b activation [27]. Whilst Murphy et al. [28] found that healthy HDL alters various PMNL functions, Curcic et al. [29] described that native HDL, in contrast to HDL modified with secretory phospholipase A2, had only minimal effects on CD11b activation and migration in PMNLs.

Activated PMNLs contribute to the chronic inflammatory state in CKD [30,31]. Whereas priming of PMNLs is an important physiologic mechanism in regulating the immune defense [32], excessive PMNL priming results in inflammation and oxidative stress [33]. Therefore, the coordinated removal of activated PMNLs via apoptosis is crucial for the termination of inflammation [15]. PMNL death is mediated by a complex network of intracellular death/survival signaling pathways and can be modulated by a variety of extracellular stimuli such as pro-inflammatory cytokines [34]. Constitutive apoptosis is regulated by intracellular signaling and changes in gene expression that define an “apoptosis differentiation program” [35]. During PMNL spontaneous apoptosis both mitochondrial- and death receptor–mediated apoptotic signaling are activated [36].

Uremic toxicity contributes to the qualitative alterations of HDL in patients with renal disease. The accumulation of the uremic toxin symmetric dimethylarginine (SDMA) in HDL of CKD patients contributes to the adverse effect of HDL in patients with impaired kidney function [37]. Post-translational modifications such as glycation and carbamylation lead to modified HDL contributing to uremic toxicity [38]. Whereas the role of advanced glycation end products as uremic toxins has been recognized for a long time [39], it is under debate if urea is a true uremic toxin [40]. However, it is responsible for carbamylation which renders HDL dysfunctional [41].

In this study we found that HDL from patients with impaired kidney function (CKD stages 3 and 4 and patients on HD treatment) significantly reduced PMNL apoptosis, a response typical of pro-inflammatory mediators [42] such as LPS or IL-18 [43]. In contrast, HDL isolated from healthy subjects did not influence PMNL apoptosis. To our knowledge, this is the first report on the effect of HDL on PMNL apoptosis.

As PMNLs exposed to the uremic milieu do not necessarily react in the same way as PMNLs from healthy subjects [44,45], we tested if HD-HDL has a different effect on PMNLs from HD patients. HD-HDL reduced apoptosis of PMNLs from HD patients to the same extent as PMNLs from healthy subjects.

Changes to the protein cargo of HDL have a significant impact on the cardioprotective properties of HDL [46]. As shown previously, HD-HDL loses its anti-inflammatory properties and can become pro-inflammatory partially as a result of SAA accumulation on the HDL particle [9,10,47]. SAA is a key acute phase protein produced by the liver under inflammatory conditions. There is a significant association between SAA levels and cardiovascular mortality in patients with high cardiovascular risk [48]. HDL-associated SAA is related to cardiac events independent of HDL-cholesterol levels [49]. Furthermore, SAA has been demonstrated to abolish the HDL-mediated attenuation of the of pro-inflammatory cytokine production by monocytes and both SAA and HDL-conjugated SAA stimulate macrophage foam cell formation [50]. However, in the present study we found that SAA significantly reduced PMNL apoptosis whereas SAA-HDL had only a small effect on PMNL morphological changes during apoptosis and no effect on the PMNL DNA content. Therefore, incorporation of SAA into HDL largely shielded its anti-apoptotic effect.

Our analyses using signal transduction inhibitors suggest that the anti-apoptotic effect of HD-HDL is based on the activation of PI3-K and ERK pathways, but not p38 MAPK. Similar results have been obtained when investigating the apoptosis attenuating influence of IL-18 and LPS which mediate pro-inflammatory effects [43].

Integrins play a crucial role in leukocyte recruitment and are involved in both the onset and resolution of inflammation [51]. CD11b mediates the adhesion of circulating PMNLs to activated endothelial cells in a first step of the pathogenesis of vascular damage [52,53] and, thus, increases atherosclerotic risk [54]. Whereas HS-HDL attenuated the fMLP-stimulated CD11b expression, and therefore exerts an anti-inflammatory effect, such a response was not observed for CKD-HDL and HD-HDL. Basal expression of CD11b was not significantly different between patient groups. Whilst significant, the effect of HS-HDL on CD11b expression was quite modest in our experimental setup. Murphy et al. [28] also reported that HDL caused a small, but insignificant, decrease in PMA-stimulated CD11b expression and reduced the PMA-stimulated CD11b activation only after an extended period of time.

In agreement with a previous study [55], treatment of PMNLs with SAA increased CD11b expression. Further, we also found that fMLP-stimulated CD11b expression was elevated by SAA, but not by HDL-associated SAA.

All tested HDL isolates showed a similar increase in fMLP-stimulated oxidative burst and had no impact in the absence of fMLP. Whilst SAA significantly increased fMLP-stimulated oxidative burst, SAA-HDL had no effect. In line with this observation, Shridas et al. [56] showed that incorporating SAA into HDL prior to cell treatment abolished SAA-mediated ROS generation and inflammasome activation. Furthermore, only lipid-poor, but not mature HDL-associated SAA is able to induce the production of pro-inflammatory cytokines in a monocyte cell line [57].

Treatment of PMNLs isolated from HD patients was stimulated by fMLP to the same degree as PMNLs from healthy subjects. However, HD-HDL did not prime PMNLs from HD patients.

Lipid rafts are plasma membrane regions that have a high cholesterol concentration and represent a platform for cell surface receptors [58]. HDL may modulate the cellular reactivity of antigen presenting cells by removing cholesterol from lipid rafts [18]. We tested the effect of MβCD, which selectively destroys lipid rafts on PMNLs [19], and found that MβCD significantly increased PMNL apoptosis. This effect was also observed in the presence of HD-HDL and was accompanied by decreased CD11b expression. This suggests that the destruction of lipid rafts counteracts the influence of anti-apoptotic factors. Interestingly, the ganglioside GM1, a lipid raft marker, disappears from PMNL surfaces early in apoptosis [59]. Furthermore, disruption of lipid rafts abrogates the delay of PMNL apoptosis by parabutoporin, a NADPH oxidase inhibitor, and therefore has a pro-apoptotic effect [60].

We found that the basal and fMLP- stimulated expression of CD11b on isolated PMNLs was increased by MβCD in a concentration dependent manner. This finding was unexpected, because it is assumed that the CD11b expression would depend on the lipid rafts integrity. Our data appear to be in contrast to a previous study [28] which showed that the disruption of lipid rafts correlates with reduced CD11b activation by PMA in whole blood. However, Solomkin et al. [61] found that treatment of PMNLs with MβCD unexpectedly caused priming that was associated with recruitment of CD11b-rich raft domains from specific granules.

Lipid rafts contribute to the assembly of active NADPH oxidase [62] and are involved in the activation of signal transduction pathways leading to the production of reactive oxygen radicals [63]. In agreement with this, we found that the destruction of lipid rafts reduced both basal oxidative burst and fMLP-mediated stimulation.

HDL from either healthy subjects or HD patients had no significant effect on PMNL chemotaxis in our under-agarose assay. This is in contrast to the results reported by Murphy et al. [28], who found an inhibitory influence of HDL from healthy subjects on PMNL chemotaxis in a transmigration assay. However, this effect was observed only after extended pre-incubation [28]. HDL spiked with SAA was also found to have no impact on PMNL chemotaxis.

The present work may have the following limitations. The HS group is less numerous than the CKD3 and 4 and HD group. Furthermore, the percentage of men in the HS, CKD3 and 4 and HD group is 44%, 75% and 67%, respectively. However, we did not observe any influence of gender on the present results and in previous studies testing PMNL functions. The CKD3 and 4 patients were significantly older than the group of HS. However, because there is no significant difference between HS and HD patients and between CKD3 and 4 patients and HD patients we believe that the age of the participants did not affect the overall conclusion of our study. Moreover, we did not see any effect of age within the groups tested.

Our data show that HDL from uremic patients affects PMNL functions such as apoptosis and CD11b expression differently from HDL of healthy subjects and thereby may contribute to systemic inflammatory characteristics of uremic patients. The pathophysiological relevance of these in vitro studies could be confirmed in in vivo experiments such as mouse models of renal failure.

## 4. Materials and Methods

### 4.1. Patients

This study was approved by the ethics committee of the General Hospital Vienna according to the declaration of Helsinki (EK 980/2011) on 1 December 2011. Informed consent was obtained from all subjects. Subjects included in this study were free of infection and intercurrent illness.

### 4.2. High-Density Lipoprotein Isolation

HDL was isolated using a one-step density gradient centrifugation as previously described [64]. Ethylene diamine tetraacetic acid (EDTA) tubes were used for blood withdrawal. The density of the plasma was adjusted to 1.24 g/mL with potassium bromide (Sigma-Aldrich, St. Louis; MO, USA). In a polyallomer centrifuge tube 4 mL of plasma was layered under phosphate-buffered saline (PBS, pH 7.4; BioWhittaker Lonza Services, Verviers, Belgium) with a density of 1.06 g/mL. After centrifugation in a fixed-angle type 75 Ti rotor in an Optima L-80 ultracentrifuge (Beckman Coulter, Fullerton, CA, USA) at 60,000 rpm (371,000× *g*) at 15 °C for 5 h, the HDL containing fraction was collected, desalted to PBS (polyacrylamide 6000 desalting column; Thermo Scientific, Rockford, IL, USA) and stored at −80 °C until further use. HDL from each group of individuals (control, CKD stage 3 and 4 and HD patients) have been tested individually.

HDL spiked with serum amyloid A (SAA-HDL) was prepared as previously described [10,65]. Fifty µg SAA (Preprotech, Rocky Hill, NL, USA) in PBS was added to 8 mL plasma from a healthy individual and incubated for 3 h at 4 °C. As control, PBS was added to 8 mL plasma from the same individual. SAA-HDL was isolated from the plasma samples as described above.

### 4.3. Polymorphonuclear Leukocyte Isolation

PMNLs were isolated from heparinized blood using discontinuous Ficoll-Hypaque (GE Healthcare Bio- Sciences AB, Uppsala, Sweden) density gradient centrifugation and hypotonic lysis of erythrocytes as previously described [66]. The viability of PMNLs obtained by this protocol was >95% as determined by ethidium bromide exclusion (GibcoBRL Life Technologies, Gaithersburg, MD, USA).

### 4.4. Inhibition Studies

The following inhibitors (Calbiochem Merck, Darmstadt, Germany) were used: SB203580 (SB), inhibitor of p38 mitogen-activated protein kinase (MAPK) at a final concentration of 30 µM, PD98059 (PD) inhibits p44/42 (extracellular-signal regulated kinase; ERK) by inhibiting MEK1,2 (MAPK kinases) at a final concentration of 50 µM, and LY294002 (LY), inhibitor of phosphoinositol 3-kinase (PI3K), at a final concentration of 50 µM. Inhibitors were dissolved in dimethyl-sulfoxide (DMSO; Sigma-Aldrich Chemie GmbH, Steinheim, Germany), with the final concentration of DMSO in assays 0.1%. At this concentration, DMSO did not affect the assays and was used as control.

### 4.5. Spontaneous PMNL Apoptosis

Spontaneous PMNL apoptosis was assessed as described in previous studies [45,67,68,69,70,71].

#### 4.5.1. Incubations

PMNLs (6 × 10^6^ cells/mL) isolated under sterile conditions were incubated at 37 °C for 20 h in PBS or in the presence of HDL at final concentrations of 10 and 100 µg/mL, as used previously [10]. All samples contained 100 U/mL penicillin–streptomycin (Gibco Life Technologies, Grand Island, NY, USA).

#### 4.5.2. Morphological Features

PMNL morphology was examined using fluorescence microscopy as previously described [67]. Cell suspensions were mixed with the fluorescent DNA-binding dyes ethidium bromide (Gibco) and acridine orange (Merck, Darmstadt, Germany) at a final concentration of 5 μg/mL each. Acridine orange binds to DNA and appears green. Ethidium bromide is taken up only by PMNLs with a damaged plasma membrane and stains DNA orange. DNA in non-apoptotic cells is structured within the nucleus and the DNA in apoptotic cells is condensed. Therefore, viable non-apoptotic (green, structured nucleus), apoptotic (green, condensed nucleus) and secondary necrotic (orange, condensed nucleus) cells can be quantified.

#### 4.5.3. DNA Content

Apoptotic cells have a lower DNA content which was analyzed by flow cytometry [72]. PMNLs (1.2 × 10^6^/200 μL) were centrifuged at 360× *g* for 20 min and washed twice with PBS. After 60 min of incubation in 250 μL ice-cold 70% ethanol on ice, the PMNLs were centrifuged and resuspended in 200 μL PBS containing 250 μg/mL RNAse (type I-A) and 50 μg/mL propidium iodide (Sigma-Aldrich Chemie GmbH, Steinheim, Germany). The samples were analyzed after 15 min at room temperature in the dark.

#### 4.5.4. Data Presentation

Apoptotic PMNLs are in a stage between viability and secondary necrosis. Under in vivo conditions, apoptotic PMNLs would be readily phagocytosed. Therefore, viable PMNLs are most important for the interpretation of our results. Our data are presented as relative viability: The value for PMNLs suspended in buffer or in the respective control was set as viability factor 1.00. Data was normalized to this value.

### 4.6. Surface CD11b Expression

Ten µL HDL of a tenfold concentrated stock solution was added to 90 µL PMNL suspension (0.3 × 10^6^ cells/mL) and incubated for 30 min at 37 °C. Then, 10 µL PBS or N-formyl-methionyl-leucyl-phenylalanine (fMLP; Sigma-Aldrich Chemie GmbH, Steinheim, Germany) stock solution (10^−7^ M) was added and incubated for another 30 min at 37 °C. After addition of a fluorescence labelled monoclonal antibody (PC5-anti-CD11b; Immunotech Beckman Coulter, Marseille, France) the samples were incubated for 45 min at room temperature and put on ice. 500 µL ice cold PBS was added. Flow cytometry was performed on an Epics XL-MCL (Coulter, Hialeah, FL, USA). The CD11b surface expression was measured as mean fluorescence intensity (MFI).

### 4.7. Oxidative Burst

#### 4.7.1. Cytochrome c Reduction

Oxidative burst was measured on isolated PMNLs via reduction of cytochrome c (Sigma-Aldrich Chemie GmbH, Steinheim, Germany) [73]. To a PMNL suspension (3.5 × 10^6^ cells/mL), cytochrome c stock solution was added (final concentration 70 µM) and incubated at 37 °C for 5 min. After addition of HDL or PBS, the incubation was continued for another 5 min. Cells were stimulated with fMLP (3.85 × 10^−7^ M final concentration), E. coli (1 to 2 × 10^8^ cells/mL final concentration) or phorbol-12-myristate-13-acetate (PMA; 1.3 × 10^−6^ M final concentration for 20 min. After centrifugation (15 min, 300× *g*, 4 °C) absorbance at 550 nm was measured.

#### 4.7.2. Whole Blood Assay

To test the effect of SAA on PMNL oxidative burst, the whole blood assay Bursttest^®^ (Opregen Pharma, Heidelberg, Germany) was used as previously described [60]. This assay is based on the conversion of dihydrorhodamine 123 to fluorescent rhodamine 123.

### 4.8. Lipid Raft Disintegration

To test the influence of selective lipid raft disintegration on PMNL functions, methyl-β-cyclodextrin (MβCD; Sigma Life Science, Sigma-Aldrich Chemie GmbH, Steinheim, Germany) was used as previously described [18].

### 4.9. Statistical Analysis

The Wilcoxon matched-pair signed-rank test was used to analyze data from at least six independent experiments. When less than six independent experiments were performed, data were analyzed by the paired two-tailed *t*-test. Data presented are mean values ± standard error of the mean (SEM).

## Figures and Tables

**Table 1:** Baseline clinical parameters of study participants.

Parameter	HS	CKD3 and 4	HD
Participants (*n*)	9	16	15
Gender (m/f)	4/5	12/4	10/5
Age (y)	45.3 (2.8)	64.2 (3.8) **	55.5 (4.5)
Total cholesterol (mg/dL)	187.5 (12.6)	186.1 (7.8)	155.3 (8.5) *^,$^
Triglycerides (mg/dL)	100.0 (10.8)	161.4 (16.9) *	166.5 (19.7) *
HDL cholesterol (mg/dL)	67.3 (5.7)	49.2 (3.4) *	39.9 (3.0) **
Albumin (g/L)	45.5 (0.8)	40.7 (1.1) **	40.0 (0.9) **
LDL cholesterol (mg/dL)	113.2 (11.5)	104.7 (7.0)	82.3 (7.3) *^,$^
Creatinine (mg/dL)	0.85 (0.07)	2.3 (0.2) **	10.03 (0.64) **^,$$^
CRP (mg/dL)	0.08 (0.02)	0.48 (0.13) *	1.19 (0.54) *

Data are shown as mean values (SEM). CKD, chronic kidney disease; CRP, C-reactive protein; HD, hemodialysis; HDL, high-density lipoprotein; HS, healthy subjects; LDL, low-density lipoprotein. * *p* < 0.05, ** *p* < 0.01 vs. HS; ^$^ *p* < 0.05, ^$$^ *p* < 0.01 vs. CKD3 and 4.

**Table 2:** Characteristics of study participants.

Parameter	HS	CKD3 and 4	HD
Participants (*n*)	9	16	15
Diabetes mellitus Type 2 (*n*)	-	4	4
Hyperlipidemia (*n*)	-	3	4
Hyperparathyroidism (*n*)	-	2	2
Osteoporosis (*n*)	-	2	2
Current medication			
ACE inhibitors (*n*)	-	3	3
Angiotensin receptor blockers (*n*)	1	10	5
β-blockers (*n*)	1	10	9
Calcium antagonists (*n*)	-	9	7
Statins (*n*)	-	7	2
Glucocorticoids (*n*)	-	2	2

CKD, chronic kidney disease; HD, hemodialysis; HS, healthy subjects; ACE, angiotensin-converting enzyme.

## References

[1] Young C.E., Karas R.H., Kuvin J.T. (2004). High-density lipoprotein cholesterol and coronary heart disease. Cardiol. Rev..

[2] Rosenson R.S., Brewer H.B., Davidson W.S., Fayad Z.A., Fuster V., Goldstein J., Hellerstein M., Jiang X.C., Phillips M.C., Rader D.J. (2012). Cholesterol efflux and atheroprotection: Advancing the concept of reverse cholesterol transport. Circulation.

[3] Wu A., Hinds C.J., Thiemermann C. (2004). High-density lipoproteins in sepsis and septic shock: Metabolism, actions, and therapeutic applications. Shock.

[4] Levine D.M., Parker T.S., Donnelly T.M., Walsh A., Rubin A.L. (1993). In vivo protection against endotoxin by plasma high density lipoprotein. Proc. Natl. Acad. Sci. USA.

[5] Barter P.J., Caulfield M., Eriksson M., Grundy S.M., Kastelein J.J., Komajda M., Lopez-Sendon J., Mosca L., Tardif J.C., Waters D.D. (2007). Effects of torcetrapib in patients at high risk for coronary events. N. Engl. J. Med..

[6] Pirillo A., Catapano A.L., Norata G.D. (2018). Biological consequences of dysfunctional HDL. Curr. Med. Chem..

[7] Marsche G., Saemann M.D., Heinemann A., Holzer M. (2013). Inflammation alters HDL composition and function: Implications for HDL-raising therapies. Pharm. Ther..

[8] Saemann M.D., Poglitsch M., Kopecky C., Haidinger M., Horl W.H., Weichhart T. (2010). The versatility of HDL: A crucial anti-inflammatory regulator. Eur. J. Clin. Investig..

[9] Tolle M., Huang T., Schuchardt M., Jankowski V., Prufer N., Jankowski J., Tietge U.J., Zidek W., van der Giet M. (2012). High-density lipoprotein loses its anti-inflammatory capacity by accumulation of pro-inflammatory-serum amyloid A. Cardiovasc. Res..

[10] Weichhart T., Kopecky C., Kubicek M., Haidinger M., Doller D., Katholnig K., Suarna C., Eller P., Tolle M., Gerner C. (2012). Serum amyloid A in uremic HDL promotes inflammation. J. Am. Soc. Nephrol..

[11] Schmitz G., Wulf G., Bruning T., Assmann G. (1987). Flow-cytometric determination of high-density-lipoprotein binding sites on human leukocytes. Clin. Chem..

[12] Blackburn W.D., Dohlman J.G., Venkatachalapathi Y.V., Pillion D.J., Koopman W.J., Segrest J.P., Anantharamaiah G.M. (1991). Apolipoprotein A-I decreases neutrophil degranulation and superoxide production. J. Lipid Res..

[13] Cohen G., Horl W.H. (2012). Immune dysfunction in uremia; an update. Toxins.

[14] Mazor R., Shurtz-Swirski R., Farah R., Kristal B., Shapiro G., Dorlechter F., Cohen-Mazor M., Meilin E., Tamara S., Sela S. (2008). Primed polymorphonuclear leukocytes constitute a possible link between inflammation and oxidative stress in hyperlipidemic patients. Atherosclerosis.

[15] Sugimoto M.A., Sousa L.P., Pinho V., Perretti M., Teixeira M.M. (2016). Resolution of Inflammation: What Controls Its Onset?. Front. Immunol..

[16] Collins J.A., Schandi C.A., Young K.K., Vesely J., Willingham M.C. (1997). Major DNA fragmentation is a late event in apoptosis. J. Histochem. Cytochem..

[17] El Kebir D., Jozsef L., Khreiss T., Pan W., Petasis N.A., Serhan C.N., Filep J.G. (2007). Aspirin-triggered lipoxins override the apoptosis-delaying action of serum amyloid A in human neutrophils: A novel mechanism for resolution of inflammation. J. Immunol..

[18] Wang S.H., Yuan S.G., Peng D.Q., Zhao S.P. (2012). HDL and ApoA-I inhibit antigen presentation-mediated T cell activation by disrupting lipid rafts in antigen presenting cells. Atherosclerosis.

[19] Sitrin R.G., Sassanella T.M., Landers J.J., Petty H.R. (2010). Migrating human neutrophils exhibit dynamic spatiotemporal variation in membrane lipid organization. Am. J. Respir. Cell Mol. Biol..

[20] Gordon S.M., Hofmann S., Askew D.S., Davidson W.S. (2011). High density lipoprotein: It’s not just about lipid transport anymore. Trends Endocrinol. Metab..

[21] Navab M., Reddy S.T., Van Lenten B.J., Fogelman A.M. (2011). HDL and cardiovascular disease: Atherogenic and atheroprotective mechanisms. Nat. Rev. Cardiol..

[22] Rye K.A., Barter P.J. (2014). Cardioprotective functions of HDLs. J. Lipid Res..

[23] Creasy K.T., Kane J.P., Malloy M.J. (2018). Emerging roles of HDL in immune function. Curr. Opin. Lipidol..

[24] Norata G.D., Pirillo A., Ammirati E., Catapano A.L. (2012). Emerging role of high density lipoproteins as a player in the immune system. Atherosclerosis.

[25] Westerterp M., Fotakis P., Ouimet M., Bochem A.E., Zhang H., Molusky M.M., Wang W., Abramowicz S., la Bastide-van Gemert S., Wang N. (2018). Cholesterol Efflux Pathways Suppress Inflammasome Activation, NETosis, and Atherogenesis. Circulation.

[26] Spirig R., Schaub A., Kropf A., Miescher S., Spycher M.O., Rieben R. (2013). Reconstituted high-density lipoprotein modulates activation of human leukocytes. PLoS ONE.

[27] Nakayama M., Nakayama K., Zhu W.J., Shirota Y., Terawaki H., Sato T., Kohno M., Ito S. (2008). Polymorphonuclear leukocyte injury by methylglyoxal and hydrogen peroxide: A possible pathological role for enhanced oxidative stress in chronic kidney disease. Nephrol. Dial. Transplant..

[28] Murphy A.J., Woollard K.J., Suhartoyo A., Stirzaker R.A., Shaw J., Sviridov D., Chin-Dusting J.P. (2011). Neutrophil activation is attenuated by high-density lipoprotein and apolipoprotein A-I in in vitro and in vivo models of inflammation. Arterioscler. Thromb. Vasc. Biol..

[29] Curcic S., Holzer M., Frei R., Pasterk L., Schicho R., Heinemann A., Marsche G. (2015). Neutrophil effector responses are suppressed by secretory phospholipase A2 modified HDL. Biochim. Biophys. Acta.

[30] Zoccali C. (2006). Traditional and emerging cardiovascular and renal risk factors: An epidemiologic perspective. Kidney Int..

[31] Yao Q., Axelsson J., Stenvinkel P., Lindholm B. (2004). Chronic systemic inflammation in dialysis patients: An update on causes and consequences. ASAIO J..

[32] Swain S.D., Rohn T.T., Quinn M.T. (2002). Neutrophil priming in host defense: Role of oxidants as priming agents. Antioxid. Redox Signal..

[33] Sela S., Shurtz-Swirski R., Cohen-Mazor M., Mazor R., Chezar J., Shapiro G., Hassan K., Shkolnik G., Geron R., Kristal B. (2005). Primed peripheral polymorphonuclear leukocyte: A culprit underlying chronic low-grade inflammation and systemic oxidative stress in chronic kidney disease. J. Am. Soc. Nephrol..

[34] Luo H.R., Loison F. (2008). Constitutive neutrophil apoptosis: Mechanisms and regulation. Am. J. Hematol..

[35] McCracken J.M., Allen L.A. (2014). Regulation of human neutrophil apoptosis and lifespan in health and disease. J. Cell Death.

[36] Daigle I., Simon H.U. (2001). Critical role for caspases 3 and 8 in neutrophil but not eosinophil apoptosis. Int. Arch. Allergy Immunol..

[37] Zewinger S., Kleber M.E., Rohrer L., Lehmann M., Triem S., Jennings R.T., Petrakis I., Dressel A., Lepper P.M., Scharnagl H. (2017). Symmetric dimethylarginine, high-density lipoproteins and cardiovascular disease. Eur. Heart J..

[38] Florens N., Calzada C., Lyasko E., Juillard L., Soulage C.O. (2016). Modified Lipids and Lipoproteins in Chronic Kidney Disease: A New Class of Uremic Toxins. Toxins.

[39] Schwenger V., Zeier M., Henle T., Ritz E. (2001). Advanced glycation endproducts (AGEs) as uremic toxins. Nahrung.

[40] Lau W.L., Vaziri N.D. (2017). Urea, a true uremic toxin: The empire strikes back. Clin. Sci..

[41] Velasquez M.T., Ramezani A., Raj D.S. (2015). Urea and protein carbamylation in ESRD: Surrogate markers or partners in crime?. Kidney Int..

[42] Geering B., Stoeckle C., Conus S., Simon H.U. (2013). Living and dying for inflammation: Neutrophils, eosinophils, basophils. Trends Immunol..

[43] Hirata J., Kotani J., Aoyama M., Kashiwamura S., Ueda H., Kuroda Y., Usami M., Okamura H., Marukawa S. (2008). A role for IL-18 in human neutrophil apoptosis. Shock.

[44] Cohen G., Raupachova J., Ilic D., Werzowa J., Horl W.H. (2011). Effect of leptin on polymorphonuclear leucocyte functions in healthy subjects and haemodialysis patients. Nephrol. Dial. Transplant..

[45] Cohen G., Raupachova J., Wimmer T., Deicher R., Horl W.H. (2008). The uraemic retention solute para-hydroxy-hippuric acid attenuates apoptosis of polymorphonuclear leukocytes from healthy subjects but not from haemodialysis patients. Nephrol. Dial. Transplant..

[46] Shao B., de Boer I., Tang C., Mayer P.S., Zelnick L., Afkarian M., Heinecke J.W., Himmelfarb J. (2015). A Cluster of Proteins Implicated in Kidney Disease Is Increased in High-Density Lipoprotein Isolated from Hemodialysis Subjects. J. Proteome Res..

[47] Han C.Y., Tang C., Guevara M.E., Wei H., Wietecha T., Shao B., Subramanian S., Omer M., Wang S., O’Brien K.D. (2016). Serum amyloid A impairs the antiinflammatory properties of HDL. J. Clin. Investig..

[48] Zewinger S., Drechsler C., Kleber M.E., Dressel A., Riffel J., Triem S., Lehmann M., Kopecky C., Saemann M.D., Lepper P.M. (2015). Serum amyloid A: High-density lipoproteins interaction and cardiovascular risk. Eur. Heart J..

[49] Kopecky C., Genser B., Drechsler C., Krane V., Kaltenecker C.C., Hengstschlager M., Marz W., Wanner C., Saemann M.D., Weichhart T. (2015). Quantification of HDL proteins, cardiac events, and mortality in patients with type 2 diabetes on hemodialysis. Clin. J. Am. Soc. Nephrol..

[50] Lee H.Y., Kim S.D., Baek S.H., Choi J.H., Cho K.H., Zabel B.A., Bae Y.S. (2013). Serum amyloid A stimulates macrophage foam cell formation via lectin-like oxidized low-density lipoprotein receptor 1 upregulation. Biochem. Biophys. Res. Commun..

[51] Kourtzelis I., Mitroulis I., von Renesse J., Hajishengallis G., Chavakis T. (2017). From leukocyte recruitment to resolution of inflammation: The cardinal role of integrins. J. Leukoc. Biol..

[52] Kaysen G.A. (2002). Role of inflammation and its treatment in ESRD patients. Blood Purif..

[53] Crockett-Torabi E., Ward P.A. (1996). The role of leukocytes in tissue injury. Eur. J. Anaesthesiol..

[54] Fardon N.J., Wilkinson R., Thomas T.H. (2001). Rapid fusion of granules with neutrophil cell membranes in hypertensive patients may increase vascular damage. Am. J. Hypertens..

[55] Badolato R., Wang J.M., Murphy W.J., Lloyd A.R., Michiel D.F., Bausserman L.L., Kelvin D.J., Oppenheim J.J. (1994). Serum amyloid A is a chemoattractant: Induction of migration, adhesion, and tissue infiltration of monocytes and polymorphonuclear leukocytes. J. Exp. Med..

[56] Shridas P., De Beer M.C., Webb N.R. (2018). High-density lipoprotein inhibits serum amyloid A-mediated reactive oxygen species generation and NLRP3 inflammasome activation. J. Biol. Chem..

[57] Kim M.H., de Beer M.C., Wroblewski J.M., Webb N.R., de Beer F.C. (2013). SAA does not induce cytokine production in physiological conditions. Cytokine.

[58] Oh H., Mohler E.R., Tian A., Baumgart T., Diamond S.L. (2009). Membrane cholesterol is a biomechanical regulator of neutrophil adhesion. Arter. Thromb. Vasc. Biol..

[59] Sheriff A., Gaipl U.S., Franz S., Heyder P., Voll R.E., Kalden J.R., Herrmann M. (2004). Loss of GM1 surface expression precedes annexin V-phycoerythrin binding of neutrophils undergoing spontaneous apoptosis during in vitro aging. Cytom. A.

[60] Remijsen Q., Vanden Berghe T., Parthoens E., Asselbergh B., Vandenabeele P., Willems J. (2009). Inhibition of spontaneous neutrophil apoptosis by parabutoporin acts independently of NADPH oxidase inhibition but by lipid raft-dependent stimulation of Akt. J. Leukoc Biol..

[61] Solomkin J.S., Robinson C.T., Cave C.M., Ehmer B., Lentsch A.B. (2007). Alterations in membrane cholesterol cause mobilization of lipid rafts from specific granules and prime human neutrophils for enhanced adherence-dependent oxidant production. Shock.

[62] Vilhardt F., van Deurs B. (2004). The phagocyte NADPH oxidase depends on cholesterol-enriched membrane microdomains for assembly. EMBO J..

[63] Lemaire-Ewing S., Lagrost L., Neel D. (2012). Lipid rafts: A signalling platform linking lipoprotein metabolism to atherogenesis. Atherosclerosis.

[64] Holzer M., Wolf P., Curcic S., Birner-Gruenberger R., Weger W., Inzinger M., El-Gamal D., Wadsack C., Heinemann A., Marsche G. (2012). Psoriasis alters HDL composition and cholesterol efflux capacity. J. Lipid Res..

[65] Mao J.Y., Sun J.T., Yang K., Shen W.F., Lu L., Zhang R.Y., Tong X., Liu Y. (2017). Serum amyloid A enrichment impairs the anti-inflammatory ability of HDL from diabetic nephropathy patients. J. Diabetes Complicat..

[66] Cohen G., Rudnicki M., Walter F., Niwa T., Horl W.H. (2001). Glucose-modified proteins modulate essential functions and apoptosis of polymorphonuclear leukocytes. J. Am. Soc. Nephrol..

[67] Cohen G., Raupachova J., Horl W.H. (2013). The uraemic toxin phenylacetic acid contributes to inflammation by priming polymorphonuclear leucocytes. Nephrol. Dial. Transplant..

[68] Cohen G., Raupachova J., Borchhardt K., Hörl W.H. (2013). Cinacalcet effect on polymorphonuclear leucocytes of kidney transplant patients. Eur. J. Clin. Investig..

[69] Wimmer T., Cohen G., Saemann M.D., Horl W.H. (2004). Effects of Tamm-Horsfall protein on polymorphonuclear leukocyte function. Nephrol. Dial. Transplant..

[70] Cohen G., Horl W.H. (2004). Retinol binding protein isolated from acute renal failure patients inhibits polymorphonuclear leucocyte functions. Eur. J. Clin. Investig..

[71] Cohen G. (2003). Immunoglobulin light chains in uremia. Kidney Int. Suppl..

[72] Riccardi C., Nicoletti I. (2006). Analysis of apoptosis by propidium iodide staining and flow cytometry. Nat. Protoc..

[73] Nauseef W.M., Metcalf J.A., Root R.K. (1983). Role of myeloperoxidase in the respiratory burst of human neutrophils. Blood.

